# Research advances of polycomb group proteins in regulating mammalian development

**DOI:** 10.3389/fcell.2024.1383200

**Published:** 2024-03-05

**Authors:** Yan Li, Yanxiang Mo, Chen Chen, Jin He, Zhiheng Guo

**Affiliations:** Department of Obstetrics and Gynecology, The First Hospital of Jilin University, Changchun, Jilin, China

**Keywords:** epigenetic modification, polycomb, PRC1, PRC2, embryo development, lineage commitment

## Abstract

Polycomb group (PcG) proteins are a subset of epigenetic factors that are highly conserved throughout evolution. In mammals, PcG proteins can be classified into two muti-proteins complexes: Polycomb repressive complex 1 (PRC1) and PRC2. Increasing evidence has demonstrated that PcG complexes play critical roles in the regulation of gene expression, genomic imprinting, chromosome X-inactivation, and chromatin structure. Accordingly, the dysfunction of PcG proteins is tightly orchestrated with abnormal developmental processes. Here, we summarized and discussed the current knowledge of the biochemical and molecular functions of PcG complexes, especially the PRC1 and PRC2 in mammalian development including embryonic development and tissue development, which will shed further light on the deep understanding of the basic knowledge of PcGs and their functions for reproductive health and developmental disorders.

## 1 Introduction

Polycomb group (PcG) genes were initially discovered in *Drosophila melanogaster* (Struhl, 1981; Nègre et al., 2006). So-called “Polycomb” refers to the extra-sex-combs phenotype observed in male flies, and the first gene in which the dominant mutation manifests as this phenotype is named *Polycomb* (*Pc*) (Kassis et al., 2017). In subsequent investigations, an increasing number of genes resembling *Polycomb* were discovered, and these genes were collectively defined as PcG genes (Kim and Kingston, 2022). It is well known that the Polycomb group (PcG) genes and their protein products are widely conserved in many animal species, from flies to humans (Kim and Kingston, 2022). Benefiting from the biochemical technologies, the definition of a PcG gene also switches from a specific mutation phenotype to the formation of PcG complexes (Piunti and Shilatifard, 2021). Currently, PcG machinery comprises two major complexes: Polycomb repressive complex 1 (PRC1) and PRC2. These two complexes can be further divided into multiple variants according to their different biochemical compositions.

Functionally, PcG complexes are generally associated with transcriptional repression (Morgan and Shilatifard, 2020). PcG complexes are originally observed to be involved in segmental determination, through repressing the expression of homeotic genes (*Hox*) (Lewis, 1978). Moreover, PcG complexes, as conserved chromatin modifiers, were found to participate in transcriptional repression of larger scale genes which are involved in the regulation of cell proliferation, stem cell pluripotency, and oncogenesis (de Potter et al., 2023). The gene silencing function of PcG complexes links to their histone-modifying activities (Morgan and Shilatifard, 2020). Notably, PRC1 mainly catalyzes the ubiquitylation of histone H2A at lysine 119, whereas PRC2 promotes the methylation of histone H3 at lysine 27 (Millán-Zambrano et al., 2022). Indeed, PcG complexes can also prevent gene expression by mediating the chromatin compaction (Piunti and Shilatifard, 2021). Beyond their roles in transcription repression, it is important to mention that PcG complexes also directly regulate gene activation in cell types dependent manner (Parreno et al., 2022).

Importantly, PcG complexes are well-known to be required for embryo development in mammals. PcG complexes mediated repressive histone modifications play an important role in silencing the transcription of inactive developmental regulator genes in early embryos, which is crucial for embryos to pass the gastrulation stage (Owen and Davidovich, 2022). In addition, PRC2-medeiated H3K27me3 controls the establishment of DNA methylation-independent imprinting which is essential for the normal development of mouse extraembryonic tissues (Chen et al., 2019). Relying on this imprinting regulation function, PcG proteins are involved in a specific process named X-chromosome inactivation (XCI). The failure of XCI in female embryos can result in developmental arrest and embryo death (Yang et al., 2016; Kobayashi, 2018). Furthermore, PcG complexes participate in the self-renewal and early lineage commitment of various tissue stem cells during development (Takano et al., 2022). A thorough understanding of polycomb protein functions is crucial to explore the molecular mechanisms underlying multiple developmental processes and developmental disease.

In this review, the general knowledge and the critical functions of polycomb proteins in mammalian embryonic development, tissue development and other biological processes were summarized and discussed.

## 2 The composition of PcG complexes

### 2.1 The composition of PRC1

The catalytic core of PRC1 is a dimer consisting of really interesting new gene 1A or B (RING1A/B) which functions as ubiquitin E3 ligases and one of the six polycomb group RING finger (PCGF) orthologs 6 which regulates PRC1 enzymatic activity (Dobrinić et al., 2021). PRC1.1-1.6, the six major groups of PRC1 complex, are defined by the different associated PCGF orthologs (Gao et al., 2012). RING1A/B and all PCGF proteins contain two conserved protein domains: Ring finger domain and WD40-associated ubiquitin-like (RAWUL) domain (Geng and Gao, 2020; de Potter et al., 2023). The similar RING domain mediates the dimerization of RING1 and PCGF, and the catalytic core of PRC1 is formed. This RING domain dimer facilitates the binding of the E2-conjugating enzyme to PRC1. Notably, PCGF2 and PCGF4 specifically comprise Proline Serine rich (PS) domains. And PCGF proteins can interact with various auxiliary subunits through their RAWUL domains (Geng and Gao, 2020; de Potter et al., 2023). Depending on the subunit associated with the RING-PCGF core, PRC1 can be further categorized as canonical PRC1 (cPRC1) and noncanonical PRC1 (ncPRC1).

cPRC1 complexes compromise RING1A/B, PCGF2/4 and one of the five chromobox (CBX) proteins (CBX2, CBX4, CBX6, CBX7 or CBX8) (Gao et al., 2012). The CBX proteins contain N-terminal chromodomains which are responsible for recruitment and stabilization of cPRC1 to specific regions of the chromatin, especially H3K27me3-rich regions. And the AT-hook of CBX proteins facilitates their binding to AT-rich major satellites DNA sequence (Bernstein et al., 2006; Gao et al., 2012). Moreover, cPRC1 also contains one of the polyhomeotic-like proteins (PHC1, PHC2 or PHC3) and sex comb on midleg homolog 1 or 2 (SCMH1/2), which facilitate the polymerization of PRC1 complexes via the sterile alpha motif (SAM) domain (Di Croce and Helin, 2013; Wani et al., 2016; Geng and Gao, 2020). Furthermore, PHC proteins can bind to the RAWUL domains of PCGF2/4 but not PCGF1/3 (Kim and Kingston, 2022).

Unlike cPRC1, the ncPRC1 complexes are made up of any of the six PCGF proteins (PCGF1-6), RING1/B and YY1 binding protein (RYBP) or its homolog YY1-associated factor 2 (YAF2) (Chan et al., 2018). RYBP and YAF2 can competitively bind to RING1B and stimulate the enzymatic activity of RING1B through a positive feedback model (Gao et al., 2012; Chagraoui et al., 2018; Liu et al., 2023). Furthermore, recent research revealed that RYBP/YAF can bind to H2AK119ub1 to further promote the spreading of H2AK119ub1 to neighbor nucleosomes (Zhao et al., 2020).

Interestingly, some subunits are found to specially exist in one of the ncPRC1-6 complexes. For example, the CXXC domain of the Lysine Demethylase 2B (KDM2B), a component of ncPRC1.1, can recognize CpG islands, and further contribute to the recruitment of ncPRC1.1 to hypomethylated CpG-rich promoters (Farcas et al., 2012; Sugishita et al., 2021). BCL6 corepressor (BCOR) and BCL6 corepressor like 1 (BCORL1) subunits are required for the stability of PCGF1 and are essential for coupling KDM2B to the enzymic core of ncPRC1.1 (Wong et al., 2016; Schaefer et al., 2022). Fibrosin (FBRS) and Autism susceptibility candidate 2 (AUTS2) are two paralog proteins of ncPRC1.3 and ncPRC1.5 (Collier et al., 2022). AUTS2 can recruit histone acetyltransferase EP300 to PRC1 complexes (Castanza et al., 2021; Pauli et al., 2021). While the role of FBRS in ncPRC1.3/5 remains elusive. Several proteins associated with PCGF6 like L3MBTL histone methyl-lysine binding protein 2 (L3MBTL2), E2F transcription factor 6 (E2F6) and MAX gene-associated protein (MGA) are common subunits of ncPRC1.6. Generally, these proteins act collaboratively to facilitate the loading of ncPRC1.6 to its target sites (Huang et al., 2018; 2022; Dahlet et al., 2021).

### 2.2 The composition of PRC2

The catalytic core of PRC2 comprises four subunits: enhancer of Zeste homologue 1/2 (EZH1/2), embryonic ectoderm development (EED), suppressor of Zeste 12 (SUZ12) and RB-binding protein 4 or 7 (RBBP4/7) (Vijayanathan et al., 2022). EZH proteins contain a SET domain, endowing PRC2 with histone lysine methyltransferase activity (Shen et al., 2008; Lee et al., 2018, 2). EED stabilizes EZH1/2 in the PRC2 and stimulates the methylation activity of EZH1/2 (Margueron et al., 2009; Hsu et al., 2020). The WD40 repeat domain (WDR) of EED is responsible for the location of H3K27me3 on PRC2 (Oksuz et al., 2018). SUZ12 functions as a structured platform interacting with other three core proteins, which contributes to the stability of the complex (Kasinath et al., 2018). WDR-containing proteins RBBP4/7 are another core component of PRC2 and modulate the recruitment of PRC2 to chromatin (Schapira et al., 2017; Huang et al., 2021).

The PRC2 core assembling with auxiliary proteins forms two distinct homocomplexes-PRC2.1 and PRC2.2 (Petracovici and Bonasio, 2021; de Potter et al., 2023). These auxiliary subunits, including Zinc finger protein AE binding protein 2 (AEBP2), Polycomb-like homologues (PCLs), PRC2-associated LCOR isoform 1 or 2 (PALI1/2), Polycomb repressive complex 2-associated protein (EPOP) and Jumonji and ATrich interaction domain containing 2 (JARID2), contain DNA binding domains which are able to recruit PRC2 to specific genomic loci and allow the adaption of PRC2 to altered cellular states (Conway et al., 2018; Huang et al., 2023). PRC2.1 is defined by the presence of one of the three PCLs (PCL1/2 PCL3) and either EPOP or PALI1/2. The C-terminal chromo-like region of PCL can directly interact with PRC2.1 core subunit SUZ12. The N-terminal domain of PCL targets the binding of PRC2.1 to specific chromatin regions (Guo et al., 2021). Interestingly, it was found that PCL proteins can bind to unmethylated DNA. The binding of the winged-helix domain of PCL1 to DNA contributes to the prolonged residence time of PCL1-PRC2 on chromatin and thereby facilitates H3K27 methylation (Choi et al., 2017). And the Tudor domains of PCL1-3 have high affinity to chromatin regions modified by H3K36me3 and give rise to the DNA-driven PRC2.1 recruitment to new target genes (Ballaré et al., 2012; Musselman et al., 2012). PCL2 can recognize and bind the regions with a high density of unmethylated CpGs in a relatively unwound helix, which is required for the precise targeting and binding of PCL2-PRC2 to the developmental genes (Perino et al., 2018). Moreover, methylated PALI1 can bind to PRC2.1-core subunit EED to allosterically activate PRC2.1 and also facilitate its DNA binding (Zhang et al., 2021). Importantly, the binding of the C-terminal domain of EPOP with the ZnB-Zn domain of SUZ12 is required for stable interactions between EPOP and PRC2.1 (Guo et al., 2021).

**FIGURE 1:**
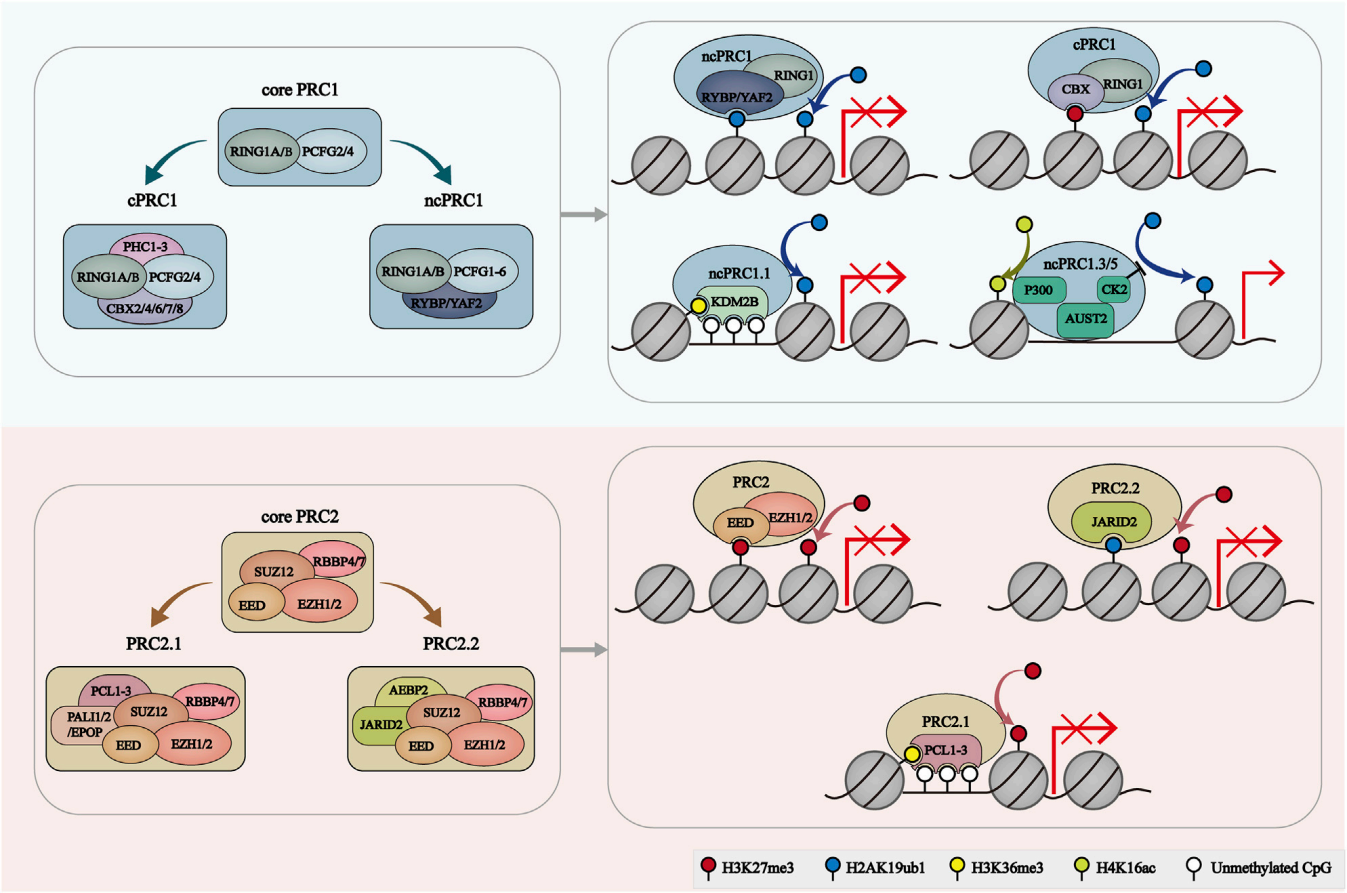
The composition and recruitment of PcG complexes. The catalytic core of PRC1 contains RING1A/B and one of six PCGF orthologs, which play a role in H2AK119ub1 deposition. PRC1 can further be divide into 2 groups: cPRC1 with RING1A/B, PCGF2/4, CBX2/4/6/7/8 and PHC1/2/3, and ncPRC1 with RING1A/B, PCGF1-6, RYBP/YAF2. RYBP/YAF2 can recognize H2AK119ub1 and facilitate the binding of ncPRC1 with chromatin. CBX proteins facilitate the cPRC1 binding to H3K27me3-deposit regions. KDM2B mediates the location of ncPRC1.1 to unmethylated CGIs. AUTS2-PRC1 is involved in transcriptional activation. PRC2 core comprises four subunits: EZH1/2, EED, SUZ12 and RBBP4/7. PRC2.1 is characterized by PCL1/2/3 and PALI1/2 or EPOP subunits. PRC2.2 was characterized by JARID2 and AEBP2 subunits. EZH2 catalyzes H3K27me3. EED, JARID2 and PCL target PRC2 to H3K27me3-enriched regions, H2AK119ub1-enriched regions and unmethylated CGIs, respectively.

Notably, PRC2.2 contains two zinc-finger-containing subunits, AEBP2 and JARID2 (Hauri et al., 2016; Kasinath et al., 2021). Both AEBP2 and JARID2 can directly bind to the ZnB-Zn domain of SUZ12 to stabilize the interactions of PRC2.2 with genomic targets (Chen et al., 2018). AEBP2 but not JARID2, competes with PCL3 to bind with the C2 domain of SUZ12 (Chen et al., 2018). JARID and EPOP share a binding domain of SUZ12 (Chen et al., 2018). Moreover, these binding modules contribute to the structural organizing of different classes of PRC2 holo complexes (Chen et al., 2018). Similar to PALI1, methylated JARID2 can also allosterically activate PRC2.2 by directly interacting with EED protein (Sanulli et al., 2015). JARID2 regulates the recruitment and activation of PRC2 by recognizing and binding H2AK119ub1-containing nucleosomes. Interestingly, cryo-electron microscopy (cryo-EM) results indicated that JARID2 and AEBP2 joint can localize to CpG-rich promoter regions with active transcription markers (H3K4me3 and H3K36me3) (Kasinath et al., 2021). JARID2 additionally contains a RNA-binding region, and the binding of JARID2 with noncoding RNAs (ncRNAs) facilitates the JARID-PRC2 interactions, which promotes the recruitment of PRC2 to chromatin (Kaneko et al., 2014) (Figure 1).

## 3 The functions of PcG complexes

The enzymatic activity of PcG complexes is essential for their function. In mammals, PRC1 regulates the deposition of H2A monoubiquitylation on K119 (H2AK119ub1), and PRC2 catalyzes mono-, di- and trimethylation of H3K27 (H3K27me2/3) (Campagne et al., 2019). These PcG-mediated histone modifications were widely considered to be associated with transcriptional repression. And PcGs can also control gene silencing by regulating 3D genomic architecture. Controversially, several researches indicated that some PcG complexes can localize at active genes and promote their expression (Campagne et al., 2019) (Figure 1).

### 3.1 PcG complexes and transcriptional repression

PcG proteins are mainly present in repressed genes which indicates their role in gene silencing. KDM2B-PRC1.1 and PCL-PRC2.1 can bind to the CGIs of silenced gene promoters and facilitate the formation of Polycomb chromatin domains which were characterized by the occupancy of H3K27me3, H2AK119ub1 as well as polycomb proteins. The interplay between PRC1 and PRC2 is necessary for the formation of polycomb domains (Dobrinić et al., 2021). In detail, H2AK119ub1 catalyzed by PRC1 can be recognized by JARID2 which recruits PRC2.2 and promotes the deposition of H3K27me3. Similarly, CBX-PRC1 can bind to the H3K27me3 and stimulate PRC1 enzymatic activity. RYBP/YAF-PRC1 and EED-PRC2 can recognize and bind to H2AK119ub1 and H3K27me3 respectively, which further promote the propagation of Polycomb domains. Furthermore, it is found that PRC2 occupancy at polycomb domains is primarily relied on the PRC1/H2AK119ub1 in mouse embryonic stem cells (Dobrinić et al., 2021). Mechanically, the occupancy of H2AK119ub1 and H3K27me3 interferes with the recruitment and activity of RNA Polymerase II (RNA Pol II), therefore largely prevents the transcription elongation by RNA Pol II. For bivalent genes with both H3K27me3 and H3K4me3, PcG proteins can hold poised RNA Pol II over the transcription start site (Flora et al., 2021). Moreover, PcG complexes can generate compacted chromatin structure, which is mainly independent on its catalytic activity. The compact state of chromatin prevents the binding of chromatin remodeling complexes such as the SWI/SNF complex, and therefore leads to transcriptional silencing. Polymerization of PRC1 which relies on SAM domains of PHC proteins is essential for polycomb-mediated chromatin structure organization (Flora et al., 2021).

### 3.2 PcG complexes and transcriptional activation

PRC1 complexes also contribute to gene activation. For instance, ncPRC1.1 subunits such as PCGF1 and KDM2B, are co-localized on actively transcribed genes (van den Boom et al., 2016). ChIP-seq data revealed the presence of a certain level of H2AK119ub1 and the absence of H3K27me3 in ncPRC1.1-bound active loci. These observations suggested that the transcriptional active role of PRC1 is independent on PRC2. Moreover, no H2AK119ub1 was detected around these loci in some cell types. In neuronal cells, AUTS2 can directly interact with histone acetyltransferase EP300 through its HX repeat domain, thus endowing ncPRC1.3/5 with the ability of transcriptional activation (Gao et al., 2014a; Castanza et al., 2021; Pauli et al., 2021). Meanwhile, AUTS2 recruits Casein kinase 2 (CK2) to phosphorylate and inactivate RING1B. As a consequence, the H2A ubiquitination activity of PRC1 was blocked (Barbour et al., 2020). A recent study further indicated that Nuclear respiratory factor 1 (NRF1) is essential for AUTS2-ncPRC1-mediated gene activation by guiding its binding to specific locus (Liu et al., 2021). There must be other factors like NRF1 that contribute to the PRC1-dependent gene activation. Therefore, how PRC1 is involved in gene activation needs to be further explored.

### 3.3 PcG complexes and X-chromosome inactivation (XCI)

XCI is a developmental process in that one of the two X chromosomes becomes silent in female cells to equalize the dosage imbalance of X-chromosome-linked genes between XY males and XX females. Mechanically, the initiation of XCI is dependent on the binding of the future inactive X chromosome (Xi) with the X-inactive specific transcript (*Xist*) which is a long noncoding RNA expressed on this chromosome in female cells (Borsani et al., 1991; Brockdorff et al., 1991; Loda et al., 2017). The *Xist* coated on Xi can recruit various complexes and allow them to bind and spread across the chromosome directly or indirectly (Markaki et al., 2021). Meanwhile, the chromatin structure of Xi is changed to a compacted state with the accumulation of DNA methylation, loss of active histone markers (like methylation of H3K4 and pan-acylation) and the deposition of repressive histone markers (like methylation of H3K9) (Keniry et al., 2016; Li et al., 2022). Furthermore, there is an accumulation of PRC1-associated H2AK119Ub and PRC2-associated H3K27me3 during this process (Brockdorff, 2017). Using female mouse embryonic stem cells, Żylicz et al. (2019) found that the enrichment of H2AK119Ub is prior to H3K27me3, and the marked regions of them are largely overlapped on the X chromosome. Interestingly, both of them are firstly deposited at the intergenic regions around the *Xist* RNA entry sites which are marked by PcGs prior to *Xist* upregulation. It seems that the initial chromatin landscape of the X chromosome instructs the alteration of the chromatin structure, which leads to the specific pattern of *Xist* spreading and transcriptional silencing (Żylicz et al., 2019). Subsequently, PcGs spread into gene bodies after deacetylation and gene silencing occurrence, which indicates that propagation of PcG-dependent markers may be not the trigger for gene repression during the XCI process (Żylicz et al., 2019). While the role of the PcG complex in XCI coordination during embryonic development *in vivo* is largely unexplored.

### 3.4 PcG complexes and genomic imprinting

In mice, a subset of imprinted genes is controlled by PRC2-mediated H3K27me3, which is termed noncanonical imprinting (Inoue, 2023). And the H3K27me3-dependant noncanonical imprinting is inherited from oocytes. Indeed, H3K27me3-mediated imprinting regulates the repression of expression of maternal *Xist*, which is responsible for protecting maternal X (Xm) from being silenced in imprinted XCI (Chen and Zhang, 2020). In autosomes, maternal H3K27me3 also contributes to maternal-specific silence of imprinted genes. After implantation, DNA methylation compensates H3K27me3 to maintain the imprinting state of these genes in the embryonic lineage. However, H3K27me3-mediated imprinting can be maintained in some genes important for placenta development in extraembryonic lineages (Chen and Zhang, 2020). While such maternal-H3K27me3 dependent noncanonical imprinting is not conserved in humans (Chen and Zhang, 2020).

## 4 H2AK119ub1 and H3K27me3 dynamics during embryonic development

H2AK119ub1 and H3K27me3, two main protein products of PcGs complexes, are largely reprogrammed after fertilization. In mouse oocytes, H2AK119ub1 and H3K27me3 are overlapped and noncanonically enriched in both promoter regions and distal regions (Chen et al., 2021). After fertilization, the H3K27me3 of promoter is largely erased, while the noncanonical maternal H3K27me3 of distal regions is inherited by zygotes (Liu et al., 2016). Moreover, the noncanonical maternal H3K27me3 is retained and controls DNA methylation-independent imprinting during preimplantation development (Chen et al., 2021; Mei et al., 2021). However, H2AK119ub1 is mainly located on the promoter regions and gene bodies of developmental related genes and plays an important role in regulating the transcription of these genes in early mouse embryos (Chen et al., 2021; Mei et al., 2021). After implantation, the distribution of H3K27me3 and H2AK119ub1 is coupled and restricted to promoter regions of developmental related genes (Chen et al., 2021; Mei et al., 2021). While maternal H3K27me3 is removed around zygotic genome activation (ZGA) in human embryos, which indicates that it unlikely to function as an imprinting marker (Xia et al., 2019; Wilkinson et al., 2023).

## 5 The role of PcG complexes in mammalian embryonic development

Embryo development is a complex process which is regulated by a series of regulators with various mechanisms. PcG proteins are responsible for mammalian embryonic development, mainly by regulating the transcriptional repression of developmental related genes. Loss of PcG proteins generally resulted in embryonic lethality after implantation. Here, we summarized and discussed the essential roles of the PcG proteins in mammalian embryo development.

### 5.1 The role of PRC1 in embryo development

#### 5.1.1 RING1B-PRC1 is responsible for embryo development

During mouse embryo development, RING1A and RING1B are not functionally redundant. *Ring1a*-null mice were viable and developed almost normally except the defects of the axial skeleton (del Mar Lorente et al., 2000). While RING1B was required for appropriate gastrulation. *Ring1b* knockout mouse embryos displayed an abnormal morphology at embryonic day 6.5 (E6.5), showing failed epiblast expansion and mesoderm migration. Finally, all *Ring1b*-null embryos were dead before E10.5 (Voncken et al., 2003). The impaired repression of *Cdkn2a* locus contributed to the early developmental arrest of *Ring1b-*null embryos (Voncken et al., 2003). It is worth noting that ablation of RING1B catalytic activity in mice and consequent loss of H2AK119ub did not affect gastrulation, and these mice were survival until E15.5 (Illingworth et al., 2015). The non-catalytic function of RING1B thus appeared to play a primary role in early embryonic development. Interestingly, RING1A and RING1B deficient embryos were arrested at the two-cell stage, accompanied by severely impaired ZGA (Posfai et al., 2012). Maternal knockout of these two genes also led to two-cell stage arrest, suggesting the maternally provided RING1B plays a major role in maternal-zygotic transition (Posfai et al., 2012). Although RING1A/B deficient oocytes completed meiosis, the dysregulated transcripts and proteins in the cytoplasm and aberrant chromatin state impaired the developmental competency of these oocytes (Posfai et al., 2012). Deletion of *Ring1a/b* at E5.5 induced embryonic lethality of both sexes at E8.5. And the female embryos exhibited more severe abnormalities than male embryos, which may be attributable to the impaired XCI in extraembryonic tissues in PRC1-null female embryos (Masui et al., 2023).

#### 5.1.2 Distinct functions of PCGF proteins

PCGF2/4 proteins are components of both cPRC1 and ncPRC1, while PCGF1/3/5/6 proteins existed only in ncPRC1 (Loh and Veenstra, 2022). Mice that lack PCGF2 or PCGF4 were viable but died after weaning, which suggests that these two proteins may compensate for each other during early embryo development (van der Lugt et al., 1994; Akasaka et al., 1996). And some similar phenotypes observed in both *Pcgf2*-null and *Pcgf4*-null mice, such as growth retardation, severe immune deficiency and posterior transformation of the axial skeleton, further supported this hypothesis (van der Lugt et al., 1994; Akasaka et al., 1996). Skeletal abnormality was likely associated with the ectopic expression of *Hox* genes (Akasaka et al., 1996). Furthermore, it was reported that PCGF2/4 is required for the maintenance but not initiation of *Hox* gene expression (Akasaka et al., 2001). Besides, *Pcgf2* mutation also resulted in neurological abnormalities characterized by ataxic gait and sporadic seizures (Akasaka et al., 1996). *Pcgf4* mutation was correlated with intestinal obstruction due to hypertrophy of intestinal smooth muscle (van der Lugt et al., 1994). These unique characteristics observed in respective null mutant mice revealed the differences in function between PCGF2 and PCGF4 at the following developmental stages (Akasaka et al., 2001).

Notably, *Pcgf1-*null mice can not develop beyond E12.5 (Dickinson et al., 2016). *Pcgf6*-knockout led to embryonic sub-lethality and the survival *Pcgf6*-null mice were fertile (Endoh et al., 2017). A subset of *Pcgf6*-null embryos was arrested as early as the blastocyst stage. *Pcgf6* knockout also induced anterior transformation of the axis and a significant reduction of placental size. These results suggest that PCGF6 is required for both pre- and post-implantation development (Endoh et al., 2017). The more pronounced developmental phenotype in *Ring1a/b* knockout embryos than that in single *Pcgf* knockout embryos indicated the necessity of all PRC1-6 proteins for embryonic development. It is worth noting that PCGF1 and PCGF6 are functionally redundant in early embryos (Mei et al., 2021). Maternal knockout of *Pcgf1/6* remarkably delayed embryonic development after the 2-cell stage, compromised implantation and reduced litter size at term (Mei et al., 2021). Further, reduced H2AK119ub1 and H3K27me3 as well as gene derepression were observed in *Pcg1/6* knockout oocytes and this abnormal epigenetic state was inherited by zygotes after fertilization. The unrepaired landscapes of H3K27me3 and H2AK119ub1 may presumably account for the embryo developmental arrest. Moreover, the enlarged placenta was also observed in PCGF1/6 maternal deficiency mice, which was caused by noncanonical imprinting loss in the extraembryonic cells (Mei et al., 2021).

Interestingly, *Pcgf3* or *Pcgf5* single knockout had a limited impact on early embryonic development (Dickinson et al., 2016; Almeida et al., 2017). Intriguingly, *Pcgf3 and Pcgf5* double knockout resulted in female-specific embryonic lethality and placental defects due to compromised *Xist*-mediated silencing (Almeida et al., 2017). Therefore, further research may be needed to illustrate how PCGF3/5-ncPRC1 interacts with the *Xist*.

#### 5.1.3 CBX and PHC proteins are indispensable for completing development

Deletion of individual CBX proteins did not impact the embryo development but resulted in different phenotypes after born. *Cbx2* knockout in mice resulted in high postnatal lethality before weaning (Coré et al., 1997). The survival mice were severely growth retarded, exhibiting skeletal malformations and male-to-female sex reversal which were associated with uncorrected expression of *Hox* genes and *Sry* respectively (Coré et al., 1997; Katoh-Fukui et al., 1998; Baumann and De La Fuente, 2011). In addition, both a hypoplastic testis and a contralateral small ovary appear in nearly 30% of CBX2-null male mice fetuses. This male-to-female sex reversal can be rescued by the forced expression of *Sry* and *Sox9* (Katoh-Fukui et al., 2012). And similar sex reversal was observed in human with XY karyotype and CBX2 mutations (Biason-Lauber et al., 2009). Furthermore, Tardat et al. (2015) found that CBX2 regulates the recruitment of PRC1 on paternal pericentric heterochromatin (pat-PCH) via its chromodomain and AT-hook. The localization of PRC1 on pat-PCH promoted the deposition of H2AK119ub which contributes to the transcriptional repression of major satellite repeats. Similarly, *Cbx4* knockout also caused abnormal embryo size and preweaning lethality in mice (Piunti and Shilatifard, 2021). While *Cbx6-*, *Cbx7-*or *Cbx8*-knockout mice can develop to adulthood. The underlying mechanism for the difference is that there are several organ defects found in CBX6-null or CBX7-null mice, but no obvious defects in CBX-8 null mice (Piunti and Shilatifard, 2021).

PHC proteins are also essential for the normal development of mice embryo. *Phc1*-null mice died during the perinatal period. While *Phc2*-null mice can survive to birth (Isono et al., 2005). And both *Phc1*-null and *Phc2*-null are involved in axial skeleton development, likely through a direct binding to the *Hoxb8* locus and repression of its transcriptional activity (Isono et al., 2005). Moreover, double knockout of *Phc1* and *Phc2* resulted in severe growth retardation and early embryonic lethality before the mid-gestational stage (Isono et al., 2005). Furthermore, *Phc3*-null animals were survived at birth but were characterized by an enlarged heart (Piunti and Shilatifard, 2021).

#### 5.1.4 KDM2B is crucial for targeting ncPRC1.1 to CpG islands

The ZF-CxxC DNA-binding domain of KDM2B can specially recognize and bind to nonmethylated CpG islands (CGIs) (Blackledge et al., 2014). With this capacity, KDM2B can target ncPRC1.1 to CGIs of inactive developmental genes, to maintain their transcriptional silencing state after implantation (Blackledge et al., 2014). Knockout of *Kdm2b* in mice caused embryonic lethality at the midgestational stage (Boulard et al., 2015). How PcG complexes bind to CpG islands and promote the deposition remains elusive. Indeed, KDM2B has two isoforms, the long isoform KDM2BLF and the short isoform KDM2BSF. It was found that KDM2BLF expression is initiated during the peri-implantation period and is decreased after E7.5, coinciding with the process of exit from naive pluripotency (Huo et al., 2022). On the one hand, KDM2BF binding depleted H3K36me2 and facilitated the deposition of H3K27me3 and H2AK119ub1 at CGIs in peri-implantation mouse embryos. On the other hand, KDM2BLF can facilitate the recruitment of BRG1/BRM-associated factor (BAF) and the core component of chromatin remodeler SWI/SNF, to the unmethylated CGIs, which led to the gain of chromatin accessibility in these regions. KDM2BLF inactivation partially compromised PcGs localization at CGIs, delayed exit from naive pluripotency and caused growth retardation as early as E6.5.

#### 5.1.5 Other components of PRC1

Mice lacking ncPRC1.6 component E2F6 were viable, with posterior homeotic transformations of the axial skeleton (Storre et al., 2002). This phenomenon is much milder than that caused by PCGF6 deletion, reflecting the redundant role of E2F6 in ncPRC1.6 during embryo development. However, E2F6 was indispensable for the repression of germline genes in preimplantation embryonic cells (Dahlet et al., 2021). Moreover, *E2f6*-knockout led to reduced DNA methylation levels in promoters of several germline genes after implantation, suggesting the responsibility of E2F6 for long-term epigenetic repression of germline genes. E2F6 functions through both PRC1-dependent and PRC1-independent mechanisms, according to the limited derepression of E2F6 targeted genes in *Pcgf6*-knockout and *Ring1a/b-knockout* ESCs (Dahlet et al., 2021).

RYBP was indispensable for the development of extraembryonic tissues, and its lack caused decidualization failure and embryonic lethality around E5.5 to E6.0. The reduced proliferation capability may contribute to embryo arrest at this stage (Pirity et al., 2005). While the role of YAF2, the homolog of RYBP, in embryo development is still largely unknown and needs further in deep investigation. However, AUTS2-null mice died before weaning, with growth retardation and defects in nervous and cardiac defects (Hori et al., 2015; Dickinson et al., 2016) (Table 1).

**TABLE 1:** The roles of PRC1 subunits in mouse development.

	Subunit	Function	Homozygous deletion phenotype	References
Core subunits	RING1A	E3 ubiquitin ligase	Anterior transformation	del Mar Lorente et al. (2000)
	RING1B		Embryonic lethal around gastrulation	Voncken et al. (2003)
cPRC1 and ncPRC1	PCGF2/4	Co-factors for H2A119ub1	Postweaning lethality	van der Lugt et al. (1994), Akasaka et al. (1996)
			Growth retardation	
			Posterior transformation	
			Immune deficiency	
cPRC1	CBX2	Recognizing and binding to H3K27me3	Preweaning lethality	Coré et al. (1997)
			Male-to-female sex reversal	
	CBX4		Preweaning lethality	Piunti and Shilatifard (2021)
	CBX6		Survival to adult	Dickinson et al. (2016)
	CBX7		Organ defects	
	CBX8		Survival to adult	
			No obvious defects	
	PHC1	polymerization of PRC1 complexes	Perinatal lethality	Isono et al. (2005)
	PHC2		Posterior transformation	
	PHC3		Enlarged heart	Dickinson et al. (2016)
ncPRC1	PCGF1	Co-factors for H2A119ub1	Can not develop beyond E12.5	Dickinson et al. (2016)
	PCGF3		Female-specific embryonic lethality	Dickinson et al. (2016), Almeida et al. (2017)
	PCGF5			
	PCGF6		Anterior transformation	Endoh et al. (2017)
	KDM2B	Recognizing and binding to unmethylated CpG islands	Embryonic lethal at midgestation	Boulard et al. (2015)
	RYBP	Stimulating the enzymatic activity of core	Embryonic lethality shortly after implantation	Pirity et al. (2005)
		Recognizing and binding to H2AK119ub1		
	AUTS2	Recruiting EP300	Pre-weaning lethality	Hori et al. (2015), Dickinson et al. (2016)

### 5.2 The role of PRC2 in embryo development

#### 5.2.1 Maternal EED and EZH are essential for embryo development

Eed is required for deposition of H3K27me3 during oogenesis in mice. Maternal EED deletion led to the loss of H3K27me3 imprinting and biallelic expression of H3K27me3-mediated imprinted genes in mouse preimplantation embryos. While absence of H3K27me3-dependent imprinting had no effect on blastocyst formation (Inoue et al., 2018; Prokopuk et al., 2018). Intriguingly, the absence of H3K27me3 imprinting resulted in death of about half *of* maternal *Eed*-knockout embryos after implantation. Those embryos showed a male-biased lethality which was already apparent by E6.5. For the live offspring, deletion of maternal *Eed* resulted in a significantly increased postnatal weight which persisted to adult life (Prokopuk et al., 2018).

*Ezh2* ablation resulted in compromised H3K27me3 establishment and embryonic arrest at the gastrulation stage (Zhao et al., 2022). The similar early lethal phenotype observed in the *Ring1b*-null, *Eed-*null and *Ezh2*-null embryos indicated the mechanistic link between PRC1 and PRC2 during gastrulation. While in contrast to the postanal overgrowth derived from oocytes lacking EED, maternal knockout of *Ezh2 or Ezh1/2* led to a significantly reduced offspring birth weight (Erhardt et al., 2003; Zhao et al., 2022). And maternal knockout of *Ezh1/2* impaired second cell lineage decision and propagation of the epiblast at the late blastocyst stage, which may be attributed to the faint H3K27me3 (Erhardt et al., 2003; Zhao et al., 2022). Notably, placental enlargement was observed in *Ezh1/2* maternal knockout embryos at E17.5, with overgrowth of the spongiotrophoblast and increased weight. The reason for the discrepancy impact of maternal EED and EZH remains unclear. And more details about the long-term effect of maternal PRC2 on offspring remain to be further explored.

#### 5.2.2 Other core subunits of PRC2

RBBP4/7 can interact with SUZ12 to guide PRC2 to target loci, facilitating the binding of PRC2 with chromosomes (Glancy et al., 2021; Mu et al., 2022). It was found that RBBP4 knockout resulted in preimplantation lethality of mouse embryos (Miao et al., 2020). While the visible H3K27me3 was observed in female blastocysts. Whether PRC2-mediated functions are comprised in RBBP4 deficiency embryos and contribute to embryo lethality needs to be further explored (Miao et al., 2020). Furthermore, loss of *Suz12* blocked embryo developmental during early postimplantation stage and induced a striking absence of H3K27me3 in embryos (Pasini et al., 2004). A significant reduction of EZH in SUZ12 knockout embryos might demonstrate that SUZ12 was essential for the stability of the EZH2 protein (Pasini et al., 2004) (Table 2).

**TABLE 2:** The roles of PRC2 subunits in mouse development.

	Subunit	Function	Homozygous deletion phenotype	References
Core subunits	EED	Recognizing and binding to H3K27me3-containing nucleosomes	Embryonic lethal around gastrulation	Inoue et al. (2018), Prokopuk et al. (2018)
	EZH1/	H3K27me3 deposition	Viable	Ezhkova et al. (2011)
	EZH2		Embryonic lethality around gastrulation	Zhao et al. (2022)
	SUZ12	Required for the stability of complex	Embryonic lethal around gastrulation	Hori et al. (2015), Dickinson et al. (2016)
	RBBP4/6	Binding to nucleosomes	Embryonic lethality around gastrulation	Miao et al. (2020)
PRC2.1	PCL2	Binding to unmethylated CpGs	Embryonic lethality around midgastrulation	Rothberg et al. (2018)
			Anemia	
PRC2.2	JARID2	Recognizing and binding H2AK119ub1-containing nucleosomes	Embryonic lethal around gastrulation	Takeuchi et al. (1995)

## 6 The role of PcG complexes in establishment and maintenance of XCI

### 6.1 EED-PRC2 is required for the establishment of XCI in preimplantation embryos

*Xist* is a maternal imprinted gene in mice. It was found that H3K27me3, but not DNA methylation, is allelic specifically deposited at the maternal *Xist* locus and contributes to its imprinting state in oocytes and early embryos (Kobayashi et al., 2012; Zheng et al., 2016; Inoue et al., 2017a; 2017b). H3K27me3-dependent imprinting of maternal *Xist* is responsible for the safeguard of the maternal X chromosome from XCI (Inoue et al., 2017b). Similar to what was found in embryos injected with *Kdm6b* at the Zygotic stage, loss of the H3K27me3 domain at the *Xist* locus led to the reactivation of maternal *Xist* and maternal XCI in maternal *Eed* knockout morula embryos (Inoue et al., 2017b). Aberrant XCI can be largely restored by E4.0 in both female and male maternal *Eed* knockout embryos, which is consistent with the results that some embryos survive to term. Specially, in female embryos, XCI occurs in a random manner in extraembryonic cells, with variable parental biases of X-linked gene expression (Inoue et al., 2018; Harris et al., 2019). Further studies are needed to clarify the mechanism underlying the conversion from Xi/Xi to Xa/Xi in *Eed* maternal KO ExEs (Inoue et al., 2018; Harris et al., 2019). And it remains to be determined whether *Xist* or autosomal H3K27me3 imprinting loss plays a major role in the male-biased lethality of EED maternal deficiency embryos. Zygotic *Eed* transcription was carried out at the 4-cell stage in mouse embryos. Different from maternal EED, the absence of zygotic EED had limited impacts on the initiation and establishment of imprinted X-inactivation but resulted in the downregulation of a subset of X-linked gens (Harris et al., 2019).

### 6.2 PcG proteins are crucial for maintenance of XCI in extraembryonic lineages

In mice, the XCI occurs with two waves. The first XCI wave occurs shortly after fertilization with a parent-of-origin bias, a systematic inactivation of the paternal X chromosome, which is called imprinted XCI (Mak et al., 2004; Okamoto et al., 2004; Ravid Lustig et al., 2023). And maternal H3K27me3 inherited from oocytes is required for the genomic imprinting of *Xist* during XCI (Inoue et al., 2017b). At the late blastocyst stage, this form of X-chromosome inactivation is reversed in cells from the inner cell mass (ICM), a process known as X-chromosome reactivation (XCR) (Patrat et al., 2009; Min et al., 2017). While the established XCI in extraembryonic lineage is maintained during the subsequent developmental stage. A random XCI (rXCI) is initiated again in embryonic lineage cells around the time of implantation. And once occurs, the status of X-inactivation will be inherited by all the progeny cells (Pacini et al., 2021).

Recently, Masui et al. (2023) induced PRCs deletion after the establishment of imprinted XCI (*Ring1a/b* deletion at E5.5 or *Eed* deletion at E3.5), and especially focused on the role of PRC1 and PRC2 in the maintenance of XCI (Borensztein et al., 2017). A disrupted suppression of *Xist*-linked genes was observed in extraembryonic lineage at E7.5, which demonstrated that PRC1/2 has a substantial impact on the maintenance of imprinted XCI in extraembryonic lineages. Notably, PRC1 and PRC2 seem to function independently since the loss of PRC1 or PRC2 did not affect the accumulation of H3K27me3 or H2AK119ub in both extraembryonic lineages and embryo at this stage (Masui et al., 2023). While previous study reported that the knockout of *Pcgf3/5* gene impaired the deposition of both H2AK119ub1 and H3K27me3 on Xi in mouse embryonic stem cells (Almeida et al., 2017). One possible reason for this discrepancy is that PRC1 may no longer be required for the propagation of PRC2 on Xi in all lineages during XCI maintenance phase (Almeida et al., 2017). In addition, the H3K27me3 accumulated on Xi during the initiation phase of XCI may sufficiently contribute to the subsequential recruitment and spread of PRC2 during the maintenance stage.

Allele-specific RNA seq revealed a partial overlap between PRC1- and PRC2-dependent X-linked genes, confirming there is a synergy between PRC1 and PRC2. More genes were sensitive to PRC1 depletion than PRC2 depletion, which implies that PRC1 is the main player in the maintenance of XCI (Masui et al., 2023). Furthermore, these PRC1-sensitive genes have a CGI at their promoter regions (Masui et al., 2023). In line with this notion, Andergassen et al. generated the PRC1 or PRC2 depleted zygotes and found that PRC2 but not PRC1 is dominant in XCI of extraembryonic tissues (Andergassen et al., 2021). The predominant impact of PRC2 on the initiation of XCI may contribute to this debate. Further studies are needed to explore the mechanisms underlying how PcGs are involved in the initiation and maintenance of XCI in mammalian embryos.

Interestingly, inconsistent with what was observed in extraembryonic tissues, the transcriptional silencing of X-linked genes still can be observed in PRC1-null or PRC2-null embryonic lineages at E7.5 (Masui et al., 2023). These results indicated that PRCs are redundant for random XCI in embryonic lineages and there must be other dominant mechanisms of XCI in embryo but not extraembryonic tissues, like DNA methylation or H3K9me3.

## 7 The role of polycomb proteins in tissue stem cells

PcG complexes are also crucial for the self-renewal and lineage commitment of stem cells. Anemia and neurological abnormalities are two characterized symptoms in survival fetuses with a deficiency of PcG proteins.

### 7.1 PcG complexes are required for hematopoiesis

PCGF1-PRC1 is essential for the balanced output of hematopoietic stem and progenitor cells (HSPCs). *Pcgf1*-deletion caused myeloid-biased differentiation of HSPCs, mainly due to the derepression of C/EBPα and *Hox* family genes (Takano et al., 2022; Nakajima-Takagi et al., 2023). Lymphoid differentiation was suppressed in *Pcgf1*-deficient cells. As a result, deletion of *Pcgf1* in hematopoietic cells led to mild anemia and leukopenia in mice (Takano et al., 2022; Nakajima-Takagi et al., 2023). Mice with a functional insufficiency BCOR that failed to interact with PCGF1 also display myeloid-biased differentiation (Tara et al., 2018). Upregulation of *Cebp* and *Hox* genes was also observed in BCOR insufficient hematopoietic cells (Tara et al., 2018). KDM2B also governs the self-renewal capacity of HSCs. KDM2B deficiency caused a significant reduction of HSPCs and compromised lymphoid specification (Andricovich et al., 2016). These results indicate that ncPRC1.1 is essential for definitive hematopoiesis and lineage commitment of HSPCs. However, PCGF4 was essential for self-renewal capacity and multipotency of HSCs through repression of the expression of cell cycle regulator INK4A/ARFPAX5 and B cell lineage developmental regulator EBF1 and PAX5 (van der Lugt et al., 1994; Oguro et al., 2010).

PRC2 also plays a pivotal role in hematopoietic development. EED is required for normal hematopoiesis during the postnatal period. Conditional knockout of *Eed* in mouse hematopoietic cells by *Vav*Cre had no visible impact on pups at birth but resulted in severe leukopenia, anemia and early lethality shortly after birth. Impaired differentiation of neonatal bone marrow (BM) hematopoiesis may account for the decreased matured blood cells (Xie et al., 2014). For adult BM HSC, loss of Eed led to HSC exhaustion which indicates EED is required for the maintenance of adult BM HSCS (Xie et al., 2014). For fetal hematopoiesis, EED loss via *Vav*Cre did not affect the development of fetal liver (FL) hematopoietic stem cells (HSCs) (Xie et al., 2014). Controversially, *EED* deletion resulted in disrupted HSC homeostasis and postimplantation lethality at mid-gestation are found in Tie2Cre (EED^CKO^) embryos (Yu et al., 2017). The earlier onset of *Tie2Cre* deletion in hemangioblasts may partially account for these different results (Yu et al., 2017).

Unlike EED, EZH2 is dispensable for the maintenance of both FL and BM HSCs (VavCre-mediated *Ezh2* excision) (Xie et al., 2014). Conversely, TieCre-mediated *EZH2* deletion resulted in largely reduced FL HSCs and embryonic lethality at the mid-gestation stage, which indicates the indispensable role of EZH2 in fetal hematopoiesis (Mochizuki-Kashio et al., 2011). It seems that EZH1 partially compensated for the deficiency of EZH2 in BM hematopoiesis, but not in fetal liver (Mochizuki-Kashio et al., 2011; Xie et al., 2014). Further, the compromised vascular integrity observed in EZH2^CKO^ embryos was lacking in EED^CKO^ embryos. EZH2 may play an important role in vascular development through EED-independent non-canonical PRC2 functions. In addition to hematopoiesis, EZH2 was reported to be required for postnatal cardiac homeostasis (Delgado-Olguín et al., 2012). Deletion of *Ezh2* in cardiac progenitors impaired postnatal cardiomyocyte differentiation and proliferation and eventually led to myocardial hypertrophy and fibrosis after birth (Delgado-Olguín et al., 2012).

SUZ12 is also required for both fetal hematopoiesis and adult HSC maintenance. SUZ12^CKO^ mice generated by VavCre died before weaning. It seems that SUZ12 may regulate fetal hematopoiesis through PRC2-independent actions. This hypothesis is further supported by the existence of a noncanonical subcomplex which contains EZH1 and SUZ12 but lacks EED (Xu et al., 2015). Moreover, lymphoid development was largely compromised in mice with a lymphoid-specific deletion of *Suz12*, displaying lymphopenia and significantly reduced spleen and thymus cellularity (Lee et al., 2015).

Unlike the core units of PRC2 expressed abundantly in all tissues, the accessory proteins are only expressed in certain tissues (Rothberg et al., 2018). The non-core units of PRC2.1-PCL2 are also found to play an important role in definitive erythroid development. The embryos lacking PCL2 were died by E15.5, displaying growth defects and anemia (Rothberg et al., 2018). *Pcl2* knockout resulted in a significantly decreased core PRC2 proteins level and a global loss of promoter H3K27me3. Mechanically, loss of PCL2-mediated H3K27me3 abnormally activated the Wnt/β-catenin signaling pathway, which resulted in impaired maturation and differentiation of erythroid (Rothberg et al., 2018) (Table 2).

### 7.2 PcG complexes are required for neuronal development

PcGs also play an essential role in the regulation of mammalian neuronal development (Desai and Pethe, 2020). For instance, *Pcgf4*-knockout in mice also caused neurological abnormalities, like ataxic gait and sporadic seizures (van der Lugt et al., 1994). Similarly, the *Auts2*-knockout induced abnormality in nervous system (Hori et al., 2015). And *Auts2*-deletion especially in the mouse central nervous system caused a phenotype similar to that of AUTS2 syndrome described in humans (Gao et al., 2014b). Contrary to the typical role of PRC1 in gene repression, AUTS2-PRC1 acts as a transcriptional activator in neuronal cells, through its recruitment of CK2 and interaction with EP300 (Gao et al., 2014b). Notably, NRF1 was required for AUTS2-PRC1 recruitment to target sites (Liu et al., 2021).

Some subunits of PRC2 are also involved in neuronal differentiation and proliferation. It was reported that EZH2 plays an important role in the fate transition of both cortical progenitor cells in the cerebral cortex and GABAergic neurons in cerebellum (Pereira et al., 2010; Feng et al., 2016). Loss of EZH2 significantly altered the timing of cortical development. Owing to the removal of H3K27me3 which was caused by *EZH2* deletion, the balance between self-renewal and differentiation of cortical progenitor cells was destroyed and turned to differentiation (Pereira et al., 2010). Cerebellar-specific deletion of *EZH2* led to an increase in cerebellar interneurons and a reduction in Purkinje cells and granule precursor cells in the embryonic cerebellum, and ultimately led to cerebellar hypoplasia (Feng et al., 2016). Moreover, PRC1 subunit Pcgf4 was also essential for cerebellar development and contributes to the expansion of granule precursor cells (Leung et al., 2004). EED regulated neuronal differentiation and proliferation of neural stem/progenitor cells. Conditional knockout of *Eed* in the brain led to postnatal lethality, with impaired neural differentiation and proliferation and malformation of the dentate gyrus (Liu et al., 2019). Overexpression of *SOX11*, the downstream target of EED, can rescue EED-ablation-induced neuronal differentiation defect. EED/PRC2-driven H3K27me1 deposition was indicated to be required for transcriptional activation of *Sox11* (Liu et al., 2019). Importantly, EED mutation in humans was related to the occurrence of Weaver syndrome which is characterized by intellectual disability (Cooney et al., 2017). Furthermore, brain malformations were also observed in mice with a heterozygous mutation of the *Suz12* gene (Miró et al., 2009).

## 8 Conclusion and outlook

Mammalian development is a continuous process which is regulated by a plenty of genes and proteins. Among them, PcG complexes are crucial for the regulation of correct development and are wildly involved in multiple biological processes, mainly including gene activation and repression, genomic imprinting, XCI and establishment of chromatin 3D structure. For precise regulation of targets, the requirements for individual PcG proteins are distinct in different developmental stages. In this review, we introduced the composition and biological functions of PcG complexes in mammals and comprehensively summarized the roles of PcG proteins in mammalian embryos and tissue stem cells. However, there are still some profound questions that are yet to be thoroughly answered.

PcGs and their catalyzed products are specially localized in developmental genes and control their expression, which is important for mammalian development. But it still remains poorly understood how polycomb proteins are recruited to their targeted genes and maintain transcriptional repression state. In mouse embryonic stem cells, PRC1/H2AK119ub1 defines the occupancy of PRC2 at Polycomb chromatin domains (Dobrinić et al., 2021). In contrast, the deposition of H2AK119ub1 is prior to H3K27me3 in early mouse embryos in promoter regions of development related genes (Chen et al., 2021; Mei et al., 2021). The recruitment model of PcG complexes needs further in deep investigation. In addition to the well-known function in repressing gene expression, it is found that PcGs are also involved in the transcriptional activation of some genes in neural cells (Liu et al., 2021). Whether PcGs act as an activator in other cell types or early embryos still need further investigation.

Although CpG proteins are well-conserved, their specialized roles demonstrated in controlling embryonic development may vary from species. For example, loss of maternal EED/PRC2 caused embryonic lethality in mice, due to the absence of noncanonical imprinting and compromised XCI. While the expression of core PRC2 genes including EED are nearly undetected (Harris et al., 2019; Lu et al., 2021). And different polycomb landscape was observed between human embryos and mouse embryos. Oocyte-specific H3K27me3 is largely retained until the blastocyst stage in mice, while it is absent after the 4-cell stage in humans and pigs (Wilkinson et al., 2023). These results indicated that H3K27me3-mediated imprinting seems only present and indispensable in rodents (Lu et al., 2021). The effects of CpG proteins on human embryo development are largely elusive. Advances in sequence technology and low-input epigenomic profiling technologies will help decipher the role of PcG complexes in human embryo development.

A recent study suggested that H3K27me3 can function as a transgenerational epigenetic carrier in *C. elegans*. The H3K27me3 state can be inherited in a Mendelian fashion, influencing the gene expression across two generations of germ cells (Kaneshiro et al., 2022). This finding indicated that the alteration of H3K27me3 state can be inherited across generations and may cause a long-term effect on the health of offspring. In mice, H3K27me3 was found to contribute to intergenerational inheritance by controlling the establishment of non-canonical imprinting (Inoue, 2023). It is largely unknown whether H3K27me3 or H2AK119ub1 contributes to the transgenerational epigenetic inheritance in mammals. Indeed, *KDM1A* overexpression can induce the reduction of mouse sperm H3K4me3 and the alteration of relative gene expression across generations (Lismer et al., 2020). Therefore, further efforts are needed to clarify the function of PcG complexes in transgenerational inheritance.

The similar protein structure between the PcG components may determines their functional redundancy, which is illustrated by the subsequent similar phenotype upon a single PcG component deficiency (Owen and Davidovich, 2022). In addition, PcG complexes have effects on each other, so the absence of one component will inevitably affect other PcG complexes. Owing to these features of PcG complexes, it is difficult to clarify the precise effects clearly in a normal state *in vivo*. Live-single molecule tracking technology will be helpful to assess the dynamic functions of CpG proteins in different developmental stages *in vivo*.
